# MaineHealth’s Comprehensive & Innovative Fall Prevention Primary Care Initiatives for Older Adults

**DOI:** 10.1093/geroni/igaf122.2636

**Published:** 2025-12-31

**Authors:** Jessica Bolduc, Maureen Higgins, Tamara Herrick

**Affiliations:** MaineHealth, Portland, Maine, United States; MaineHealth, Portland, Maine, United States; MaineHealth, Portland, Maine, United States

## Abstract

Falls are a major concern for adults over 65, with one in four experiencing a fall each year—approximately 14 million falls and 39,000 fall-related deaths nationwide. In Maine alone, over 100,000 falls were recorded in 2023 and 320 fall-related deaths in 2024. The U.S. incurs over $80 billion annually in costs associated with fall-related care for older adults. Maine ranks among the highest in the United States for fall rates and related fatalities and spends over $255 million annually. Recognizing the urgency of this issue, MaineHealth, the largest integrated health system in Northern New England, is implementing innovative and comprehensive fall prevention strategies. Using a multifaceted approach that integrates education, technology, and community engagement, these initiatives equip both care teams and patients with essential tools and resources. Embedding the Age-Friendly 4M framework alongside the CDC’s STEADI initiative, MaineHealth enhances fall risk screening, assessment, and intervention through EMR integration to facilitate streamlined care delivery. MaineHealth is building infrastructure to provide A Matter of Balance: Managing Concerns about Falls-Virtual, an evidence-based fall prevention program proven to interrupt the cycle of fear of falling and increase activity levels, in order to increase access to older adults that screen at risk for falls throughout the healthcare system. Early outcomes from these efforts in ambulatory care demonstrate feasibility and ongoing opportunities for expansion. Positioned at the intersection of public health and clinical care, MaineHealth is committed to collaborative solutions to achieve our goal of working together so our communities are the healthiest in America.

